# Supplementary material for: Microbiome succession during ammonification in eelgrass bed sediments
Source: PeerJ. 2017 Aug 16;5:e3674. doi: 10.7717/peerj.3674 (PMC5563154; doi:10.7717/peerj.3674)
Supplement: Table S1 — We used Kruskal–Wallis tests with 9999 permutations to assess whether alpha diversity was significantly different between categories. We used four different measurements of alpha diversity (observed number of OTUs, Chao1, Shannon Inverse Simpson). Categories examined included timepoint, eelgrass status (one genotype, multiple genotypes or none present), eelgrass initial relatedness (low, medium, high), eelgrass final richness and plot location. [file peerj-05-3674-s001.docx]

| **Category** | **Metric** | **Chi-squared** | **P (perm)** |
| --- | --- | --- | --- |
| **Timepoint** | Observed number of OTUs | 80.406 | < 0.001 |
|  | Chao1 | 69.659 | < 0.001 |
|  | Shannon | 140.6 | < 0.001 |
|  | Inverse Simpson | 121.36 | < 0.001 |
| **Eelgrass Status** | Observed number of OTUs | 0.72 | 0.7 |
|  | Chao1 | 0.618 | 0.73 |
|  | Shannon | 0.679 | 0.72 |
|  | Inverse Simpson | 1.344 | 0.506 |
| **Eelgrass richness** | Observed number of OTUs | 6.322 | 0.383 |
|  | Chao1 | 7.057 | 0.313 |
|  | Shannon | 3.488 | 0.755 |
|  | Inverse Simpson | 4.598 | 0.595 |
| **Eelgrass relatedness** | Observed number of OTUs | 1.672 | 0.433 |
|  | Chao1 | 1.488 | 0.468 |
|  | Shannon | 0.688 | 0.705 |
|  | Inverse Simpson | 1.083 | 0.582 |
| **Plot location** | Observed number of OTUs | 62.161 | 0.797 |
|  | Chao1 | 69.518 | 0.543 |
|  | Shannon | 45.346 | 0.997 |
|  | Inverse Simpson | 42.76 | 0.998 |
