# Supplementary material for: Microbiome succession during ammonification in eelgrass bed sediments
Source: PeerJ. 2017 Aug 16;5:e3674. doi: 10.7717/peerj.3674 (PMC5563154; doi:10.7717/peerj.3674)
Supplement: Table S2 — Alpha diversity was determined to be significantly different across timepoints (Table 1). We examined which timepoint comparisons were stochastically dominant using the Dunn test on four different measurements of alpha diversity (observed number of OTUs, Chao1, Shannon Inverse Simpson). Timepoint 1 (initial samples), 2 (seven days), 3 (13 days), and 4 (19 days). [file peerj-05-3674-s002.docx]

| **Metric** | **Pairwise comparison** | **Z score** | **p-value** | **Bonferroni corrected p-value** |
| --- | --- | --- | --- | --- |
| **Observed number of OTUs** | 1 - 2 | 5.276 | < 0.001 | < 0.001 |
|  | 1 - 3 | 8.874 | < 0.001 | < 0.001 |
|  | 2 - 3 | 3.598 | < 0.001 | .002 |
|  | 1 - 4 | 5.410 | < 0.001 | < 0.001 |
|  | 2 - 4 | 0.134 | 0.094 | 1 |
|  | 3 - 4 | -3.464 | < 0.001 | .003 |
| **Chao1** | 1 - 2 | 4.540 | < 0.001 | < 0.001 |
|  | 1 - 3 | 8.335 | < 0.001 | < 0.001 |
|  | 2 - 3 | 3.795 | < 0.001 | < 0.001 |
|  | 1 - 4 | 4.308 | < 0.001 | < 0.001 |
|  | 2 - 4 | -0.232 | 0.816 | 1 |
|  | 3 - 4 | -4.027 | < 0.001 | < 0.001 |
| **Shannon** | 1 - 2 | 8.073 | < 0.001 | < 0.001 |
|  | 1 - 3 | 9.503 | < 0.001 | < 0.001 |
|  | 2 - 3 | 1.430 | 0.153 | 0.917 |
|  | 1 - 4 | 10.730 | < 0.001 | < 0.001 |
|  | 2 - 4 | 2.657 | 0.008 | 0.047 |
|  | 3 - 4 | 1.227 | 0.220 | 1 |
| **Inverse Simpson** | 1 - 2 | 8.210 | < 0.001 | < 0.001 |
|  | 1 - 3 | 7.842 | < 0.001 | < 0.001 |
|  | 2 - 3 | -0.368 | 0.713 | 1 |
|  | 1 - 4 | 10.204 | < 0.001 | < 0.001 |
|  | 2 - 4 | 1.994 | 0.0461 | 0.277 |
|  | 3 - 4 | 2.362 | 0.0182 | 0.109 |
