# Supplementary material for: Microbiome succession during ammonification in eelgrass bed sediments
Source: PeerJ. 2017 Aug 16;5:e3674. doi: 10.7717/peerj.3674 (PMC5563154; doi:10.7717/peerj.3674)
Supplement: Table S3 — PERMANOVA tests were performed to find significant differences in microbial beta diversity, calculated as the Weighted Unifrac distance metric, between different categorical variables including initial plot treatment (number of genotypes × level related), eelgrass plot richness, eelgrass initial level related (low, medium, high), eelgrass genotypic evenness, eelgrass status (one genotype, multiple genotypes or none present), timepoint, block (A–L), eelgrass richness, spot (1–6) and plot location (block × spot). [file peerj-05-3674-s003.docx]

| **Category** | **Pseudo-F** | **R2** | **p-value** | **Bonferroni Corrected p-value** |
| --- | --- | --- | --- | --- |
| Plot treatment (initial) | 0.921 | 0.017 | 0.567 | 1 |
| Eelgrass richness (initial) | 1.234 | 0.004 | 0.235 | 1 |
| Eelgrass level related (initial) | 0.727 | 0.005 | 0.740 | 1 |
| Eelgrass genotypic evenness | 0.929 | 0.135 | 0.707 | 1 |
| Eelgrass status | 0.797 | 0.006 | 0.643 | 1 |
| Timepoint | 22.551 | 0.197 | < 0.001 | 0.004 |
| Block | 1.560 | 0.060 | 0.006 | 0.274 |
| Eelgrass richness (final) | 0.869 | 0.019 | 0.675 | 1 |
| Spot | 1.109 | 0.020 | 0.285 | 1 |
| Plot location | 0.903 | 0.236 | 0.918 | 1 |
