# Supplementary material for: Microbiome succession during ammonification in eelgrass bed sediments
Source: PeerJ. 2017 Aug 16;5:e3674. doi: 10.7717/peerj.3674 (PMC5563154; doi:10.7717/peerj.3674)
Supplement: Table S4 — Comparing microbial community structure between pair-wise timepoints using multiple beta diversity metrics (Weighted Unifrac, Unweighted Unifrac, Bray Curtis) to assess at which timepoints, the communities differed significantly. Timepoint 1 (initial samples), 2 (seven days), 3 (13 days), and 4 (19 days). [file peerj-05-3674-s004.docx]

| **Metric** | **Pairwise comparison** | **Pseudo-F** | **R2** | **P (perm)** | **Bonferroni corrected P** |
| --- | --- | --- | --- | --- | --- |
| **Weighted Unifrac** | 1 - 2 | 33.511 | 0.191 | < 0.001 | < 0.001 |
|  | 2 - 3 | 7.083 | 0.049 | < 0.001 | < 0.001 |
|  | 1 - 3 | 26.547 | 0.162 | < 0.001 | < 0.001 |
|  | 2 - 4 | 21.879 | 0.136 | < 0.001 | < 0.001 |
|  | 1 - 4 | 36.924 | 0.210 | < 0.001 | < 0.001 |
|  | 3 - 4 | 13.074 | 0.089 | < 0.001 | < 0.001 |
| **Unweighted Unifrac** | 1 - 2 | 3.832 | 0.026 | < 0.001 | < 0.001 |
|  | 2 - 3 | 2.886 | 0.021 | < 0.001 | < 0.001 |
|  | 1 - 3 | 7.047 | 0.049 | < 0.001 | < 0.001 |
|  | 2 - 4 | 3.372 | 0.024 | < 0.001 | < 0.001 |
|  | 1 - 4 | 7.322 | 0.050 | < 0.001 | < 0.001 |
|  | 3 - 4 | 1.891 | 0.014 | < 0.001 | < 0.001 |
| **Bray Curtis** | 1 - 2 | 12.500 | 0.081 | < 0.001 | < 0.001 |
|  | 2 - 3 | 3.289 | 0.023 | < 0.001 | < 0.001 |
|  | 1 - 3 | 13.449 | 0.089 | < 0.001 | < 0.001 |
|  | 2 - 4 | 12.608 | 0.083 | < 0.001 | < 0.001 |
|  | 1 - 4 | 20.003 | 0.126 | < 0.001 | < 0.001 |
|  | 3 - 4 | 7.494 | 0.053 | < 0.001 | < 0.001 |
