# Supplementary material for: Microbiome succession during ammonification in eelgrass bed sediments
Source: PeerJ. 2017 Aug 16;5:e3674. doi: 10.7717/peerj.3674 (PMC5563154; doi:10.7717/peerj.3674)
Supplement: Table S6 — Mantel tests were used to identify significant correlations between microbial beta diversity, calculated as Bray Curtis dissimilarities, and different quantitative variables including ammonification rate (µmol NH4-N/L sediment/d), total belowground biomass (g/plot), total aboveground biomass (g/plot) and total biomass (g/plot). [file peerj-05-3674-s006.docx]

| **Environmental variable** | **Mantel r statistic** | **p-value** |
| --- | --- | --- |
| Ammonification rate | -0.036 | 0.836 |
| Total belowground biomass | 0.014 | 0.705 |
| Total aboveground biomass | 0.003 | 0.408 |
| Total biomass | -0.006 | 0.586 |
