# Supplementary material for: Microbiome succession during ammonification in eelgrass bed sediments
Source: PeerJ. 2017 Aug 16;5:e3674. doi: 10.7717/peerj.3674 (PMC5563154; doi:10.7717/peerj.3674)
Supplement: Table S7 — Mantel tests were used to identify significant correlations between microbial beta diversity, calculated as Bray Curtis dissimilarities, and different quantitative variables at timepoint #1. The quantitative variables tested include ammonification rate (µmol NH4-N/L sediment/d), total belowground biomass (g/plot), total aboveground biomass (g/plot), total biomass (g/plot), rhizome biomass (g/plot), root biomass (g/plot), Rao’s Q, eelgrass genotypic evenness, eelgrass Shannon Diversity, eelgrass average relatedness, plot detritus standing stock (g/plot) from prior months (June, July, August) and plot decomposition rate. [file peerj-05-3674-s007.docx]

| **Variable** | **Mantel r statistic** | **p-value** |
| --- | --- | --- |
| Ammonification rate | 0.042 | 0.258 |
| Total belowground biomass | 0.042 | 0.200 |
| Total aboveground biomass | 0.034 | 0.188 |
| Total biomass | 0.039 | 0.180 |
| Rhizome biomass | 0.063 | 0.103 |
| Root biomass | -0.006 | 0.530 |
| Rao’s Q | 0.033 | 0.273 |
| Eelgrass genotypic evenness | -0.023 | 0.689 |
| Eelgrass Shannon Diversity | 0.045 | 0.212 |
| Eelgrass average relatedness | -0.021 | 0.678 |
| June detritus standing stock | - 0.0523 | 0.096 |
| July detritus standing stock | -0.096 | 0.948 |
| August detritus standing stock | - 0.062 | 0.168 |
| Plot decomposition rate | - 0.004 | 0.471 |
