# Supplementary material for: Microbiome succession during ammonification in eelgrass bed sediments
Source: PeerJ. 2017 Aug 16;5:e3674. doi: 10.7717/peerj.3674 (PMC5563154; doi:10.7717/peerj.3674)
Supplement: Table S8 — The average relative abundance of taxonomic orders was compared between timepoints using Bonferroni corrected Kruskal–Wallis tests. [file peerj-05-3674-s008.docx]

| **Order** | **Chi-squared** | **p-value** | **Bonferroni corrected p-value** |
| --- | --- | --- | --- |
| Pirellulales | 24.196 | < 0.001 | < 0.001 |
| GCA004 | 9.462 | 0.024 | 0.214 |
| Chromatiales | 55.328 | < 0.001 | < 0.001 |
| Desulfobacterales | 45.850 | < 0.001 | < 0.001 |
| Flavobacteriales | 14.615 | 0.002 | 0.020 |
| Bacteroidales | 88.955 | < 0.001 | < 0.001 |
| Alteromonadales | 32.819 | < 0.001 | < 0.001 |
| Campylobacterales | 102.708 | < 0.001 | < 0.001 |
| Thiotrichales | 39.869 | < 0.001 | < 0.001 |
