# Supplementary material for: Microbiome succession during ammonification in eelgrass bed sediments
Source: PeerJ. 2017 Aug 16;5:e3674. doi: 10.7717/peerj.3674 (PMC5563154; doi:10.7717/peerj.3674)
Supplement: Table S9 — Post-hoc Dunn tests were were performed on taxonomic orders that were found to have significantly different mean relative abundances across timepoints using Kruskal–Wallis tests (Table S8). These tests were used to identify which timepoint comparisons showed stochastic dominance. Only sequential timepoint comparisons are shown here. Timepoint 1 (initial samples), 2 (seven days), 3 (13 days), and 4 (19 days). [file peerj-05-3674-s009.docx]

| **Order** | **Pairwise comparison** | **Z score** | **p-value** | **Bonferroni corrected p-value** |
| --- | --- | --- | --- | --- |
| **Thiotrichales** | 1 - 2 | 3.182 | 0.001 | 0.009 |
|  | 2 - 3 | -1.347 | 0.178 | 1 |
|  | 3 - 4 | -5.443 | < 0.001 | < 0.001 |
| **Campylobacterales** | 1 - 2 | -9.842 | < 0.001 | < 0.001 |
|  | 2 - 3 | -5.666 | < 0.001 | < 0.001 |
|  | 3 - 4 | 1.295 | 0.195 | 1 |
| **Alteromonadales** | 1 - 2 | -0.462 | 0.644 | 1 |
|  | 2 - 3 | -5.138 | < 0.001 | < 0.001 |
|  | 3 - 4 | -3.997 | < 0.001 | < 0.001 |
| **Bacteroidales** | 1 - 2 | 4.388 | < 0.001 | < 0.001 |
|  | 2 - 3 | 8.998 | < 0.001 | < 0.001 |
|  | 3 - 4 | 2.218 | 0.027 | 0.160 |
| **Flavobacteriales** | 1 - 2 | -3.394 | < 0.001 | 0.004 |
|  | 2 - 3 | -0.343 | 0.732 | 1 |
|  | 3 - 4 | 1.573 | 0.116 | 0.694 |
| **Desulfobacterales** | 1 - 2 | 4.857 | < 0.001 | < 0.001 |
|  | 2 - 3 | 6.503 | < 0.001 | < 0.001 |
|  | 3 - 4 | 2.755 | 0.006 | 0.035 |
| **Chromatiales** | 1 - 2 | 3.030 | 0.002 | 0.015 |
|  | 2 - 3 | 6.715 | < 0.001 | < 0.001 |
|  | 3 - 4 | 0.863 | 0.388 | 1 |
| **Pirellulales** | 1 - 2 | 3.699 | < 0.001 | 0.001 |
|  | 2 - 3 | 3.826 | < 0.001 | < 0.001 |
|  | 3 - 4 | 3.190 | 0.001 | 0.009 |
|  |  |  |  |  |
