# Supplementary material for: Microbiome succession during ammonification in eelgrass bed sediments
Source: PeerJ. 2017 Aug 16;5:e3674. doi: 10.7717/peerj.3674 (PMC5563154; doi:10.7717/peerj.3674)
Supplement: Table S10 — Only orders with a mean relative abundance of greater than or equal to 2 percent are show here. [file peerj-05-3674-s010.docx]

| Timepoint | Phylum | Class | Order | Mean | Standard deviation | Standard error |
| --- | --- | --- | --- | --- | --- | --- |
| 1 | Bacteroidetes | Bacteroidia | Bacteroidales | 8.248 | 1.558 | 0.184 |
| 2 | Bacteroidetes | Bacteroidia | Bacteroidales | 6.848 | 1.537 | 0.181 |
| 3 | Bacteroidetes | Bacteroidia | Bacteroidales | 6.173 | 1.871 | 0.229 |
| 4 | Bacteroidetes | Bacteroidia | Bacteroidales | 5.110 | 1.900 | 0.229 |
| 1 | Bacteroidetes | Flavobacteriia | Flavobacteriales | 6.479 | 1.389 | 0.164 |
| 2 | Bacteroidetes | Flavobacteriia | Flavobacteriales | 7.416 | 1.405 | 0.166 |
| 3 | Bacteroidetes | Flavobacteriia | Flavobacteriales | 6.883 | 1.521 | 0.186 |
| 4 | Bacteroidetes | Flavobacteriia | Flavobacteriales | 6.236 | 2.530 | 0.305 |
| 1 | Chloroflexi | Anaerolineae | GCA004 | 4.259 | 1.002 | 0.118 |
| 2 | Chloroflexi | Anaerolineae | GCA004 | 4.642 | 1.081 | 0.127 |
| 3 | Chloroflexi | Anaerolineae | GCA004 | 4.405 | 1.158 | 0.141 |
| 4 | Chloroflexi | Anaerolineae | GCA004 | 3.878 | 1.808 | 0.218 |
| 1 | Planctomycetes | Planctomycetia | Pirellulales | 2.219 | 0.412 | 0.048 |
| 2 | Planctomycetes | Planctomycetia | Pirellulales | 1.924 | 0.439 | 0.052 |
| 3 | Planctomycetes | Planctomycetia | Pirellulales | 2.193 | 0.550 | 0.067 |
| 4 | Planctomycetes | Planctomycetia | Pirellulales | 1.825 | 0.742 | 0.089 |
| 1 | Proteobacteria | Deltaproteobacteria | Desulfobacterales | 8.354 | 1.054 | 0.124 |
| 2 | Proteobacteria | Deltaproteobacteria | Desulfobacterales | 7.291 | 0.997 | 0.117 |
| 3 | Proteobacteria | Deltaproteobacteria | Desulfobacterales | 7.456 | 1.449 | 0.177 |
| 4 | Proteobacteria | Deltaproteobacteria | Desulfobacterales | 6.346 | 2.383 | 0.287 |
| 1 | Proteobacteria | Epsilonproteobacteria | Campylobacterales | 4.801 | 2.199 | 0.259 |
| 2 | Proteobacteria | Epsilonproteobacteria | Campylobacterales | 12.570 | 5.916 | 0.697 |
| 3 | Proteobacteria | Epsilonproteobacteria | Campylobacterales | 9.362 | 3.461 | 0.423 |
| 4 | Proteobacteria | Epsilonproteobacteria | Campylobacterales | 8.650 | 4.029 | 0.485 |
| 1 | Proteobacteria | Gammaproteobacteria | Alteromonadales | 3.08 | 0.505 | 0.060 |
| 2 | Proteobacteria | Gammaproteobacteria | Alteromonadales | 3.118 | 0.582 | 0.069 |
| 3 | Proteobacteria | Gammaproteobacteria | Alteromonadales | 3.601 | 1.874 | 0.229 |
| 4 | Proteobacteria | Gammaproteobacteria | Alteromonadales | 6.214 | 4.922 | 0.593 |
| 1 | Proteobacteria | Gammaproteobacteria | Chromatiales | 7.356 | 1.069 | 0.126 |
| 2 | Proteobacteria | Gammaproteobacteria | Chromatiales | 6.658 | 1.344 | 0.158 |
| 3 | Proteobacteria | Gammaproteobacteria | Chromatiales | 5.912 | 1.344 | 0.164 |
| 4 | Proteobacteria | Gammaproteobacteria | Chromatiales | 5.564 | 1.674 | 0.201 |
| 1 | Proteobacteria | Gammaproteobacteria | Thiotrichales | 9.743 | 1.333 | 0.157 |
| 2 | Proteobacteria | Gammaproteobacteria | Thiotrichales | 8.803 | 1.666 | 0.196 |
| 3 | Proteobacteria | Gammaproteobacteria | Thiotrichales | 9.361 | 4.830 | 0.590 |
| 4 | Proteobacteria | Gammaproteobacteria | Thiotrichales | 18.525 | 13.858 | 1.668 |
